# Anesthetic key points in a patient with a terminal ileum neuroendocrine tumor and a rare carcinoid left heart disease presented for non-cardiac surgery: case report

**DOI:** 10.1186/s12871-024-02648-w

**Published:** 2024-07-31

**Authors:** Kevin Van Ussel, Daniel Leonard, Christine Watremez, Cristina Bianca Robu

**Affiliations:** 1 Department of Anesthesiology, Cliniques Universitaires Saint Luc, Université Catholique de Louvain, Av Hippocrate 10, Brussels, 1200 Belgium; 2 Department of Colorectal Surgery, Cliniques Universitaires Saint Luc, Université Catholique de Louvain, Brussels, Belgium; 3 Brussels, Belgium

**Keywords:** Carcinoid tumors, Carcinoid syndrome, Carcinoid heart disease, Somatostatin, Octreotide, Neuroendocrine tumor, Anesthetic management

## Abstract

**Background:**

Carcinoid tumors are rare neuroendocrine malignancies presenting in an increasing number in our center. The incidence of carcinoid tumors is approximatively between 2.5 and 5 cases per 100,000 people of whom about 50% develop carcinoid syndrome. Once the carcinoid syndrome has developed, a carcinoid cardiomyopathy can occur. Carcinoid heart disease (CaHD) remains a serious and rare complication associated with a significant increase in morbidity and mortality. Although carcinoid tumors have been known and studied for several years, there are still scarce data on the anesthetic management and the peri operative period.

**Case presentation:**

We describe a case of a Caucasian 44-year-old woman with an unusual presentation of left CaHD with an ileal neuroendocrine tumor and liver metastases. Our preoperative somatostatin administration protocol, limit the cardiac damage. The maintenance of stable hemodynamics, the use of balanced anesthetic technique, all along with a good understanding of the pathology, played a major role in the successful management of anesthesia. This case report allows us to introduce our decision algorithm for the management of this type of pathology in our tertiary hospital, Cliniques Universitaires Saint-Luc.

**Conclusion:**

Despite the paucity of data, anesthetic management of patients with carcinoid tumor can be safely performed with effective hemodynamic monitoring and a good understanding of the pathophysiology. Knowledge and application of a clear institutional algorithm for octreotide administration and multidisciplinary consultation at a referral center are essential for the management of these patients.

**Supplementary Information:**

The online version contains supplementary material available at 10.1186/s12871-024-02648-w.

## Background

Neuroendocrine tumors (NET) are rare cancers that develop in the specialized cells of body’s neuroendocrine system, the incidence of carcinoid tumors is approximatively between 2.5 and 5 cases per 100,000 people [1, 2], they represent 0.49% of all malignant tumors encountered. As a NET often causes no symptoms, about 15% of patients present liver metastases at the time of diagnosis [1–4] and approximatively 50% of patient develop symptoms of carcinoid syndrome [5, 6]. The tumor and the metastases can produce and secrete a variety of vasoactive substances (mainly serotonin or histamine) which cause the symptoms that define the carcinoid syndrome (CaS) [1, 2].

The carcinoid heart disease (CaHD) is a consequence of the carcinoid syndrome which typically causes abnormalities of the valves [7]. It can occur in up to 50% of patients suffering from CaS [6, 8] and in 20% of the cases the CaHD could be the first clinical manifestation. CaHD is a serious complication of the CaS associated with a significantly increased morbidity and mortality rate [8, 9]. Three-year survival in the presence of carcinoid heart disease is 31% versus 68% for patients with neuroendocrine metastases without carcinoid heart disease [10, 11]. Plaque deposition classically occurs in the endocardium and valve leaflets but can also occur in the chordae tendineae. These deposits mainly affect the right heart (in 90% of cases). The left heart is less affected due to inactivation in the lung of tumor secreted vasoactive substances [5, 12]. The presence of an intracardiac shunt (such as atrial septal defect or foramen ovale), pulmonary metastases or high tumor activity [7, 13] are some causes that affect preferably the left heart.

## Case report

We report a case of a 44-year-old woman, 69 kg for 1.76 m height, with a NET located in the terminal ileum and liver metastases who presented a CaS with a left sided CaHD. She was admitted at the hospital with class IV New York Heart association (NYHA) dyspnea and orthopnea due to severe mitral and aortic valve insufficiency. The patient also complained of non-specific abdominal pain. No flushing, tachycardia or diarrhea was reported. Upon physical examination, a grade 3/6 systolic and 3/6 diastolic murmur was identified. Electrocardiography showed sinus rhythm with left atrial and ventricular hypertrophy. Transthoracic echocardiography (TTE) and trans-esophageal echocardiography (TEE) showed left ventricle dilation with preserved global systolic function, a severe central jets of mitral regurgitation, a severe aortic incompetence (Image 1.) and a severe pulmonary hypertension (PH) with a moderate tricuspid insufficiency and a systolic PAP at 69 mmHg. The pulmonary function tests were normal. Upon admission, the blood results showed a NT-pro BNP level increased at 1549 pg/mL. The other laboratory parameters did not show any abnormalities. No degradation product of serotonin were measured at that time. The diagnosis was made following the positron emission tomography (PET-CT) and colonoscopy carried out in the presence of nonspecific symptomatology. Abdominal computer tomography confirmed a terminal ileum neoplasm, identified as the primary tumor with limited liver metastatic disease. Suspicion of CaS with left-sided CaHD secondary to NET of terminal ileum was raised and the patient was medicated with somatostatin (120 mg 1x/4 weeks) to provide relief of symptoms. Moreover, upon admission, and given the cardiac symptomatology, a treatment by inhibitor of ACE and diuretics was initiated. In this context surgery played a vital role in the treatment of the CaS, hence, the case was discussed in a multidisciplinary team. The indication for valvular surgery was discussed but given of the early oncological status of the primary lesion this strategy was not retained.

**Fig. 1:**
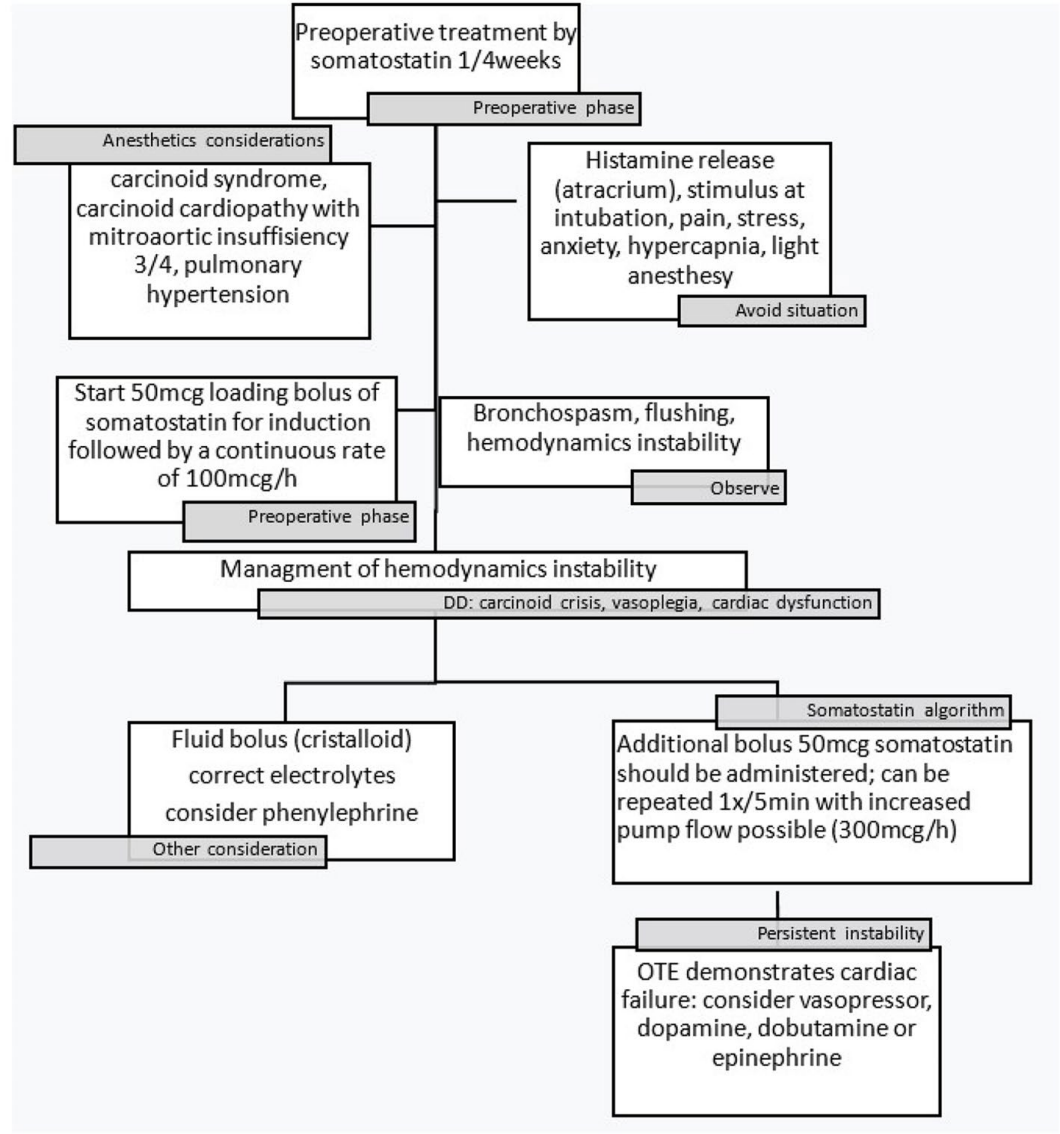
Algorithm for management of carcinoid syndrome and carcinoid heart disease

Terminal ileum resection by laparoscopy was declined for the safety reasons. Therefore, an open abdominal surgery was approved and scheduled. The surgery was conducted with the patient under general anesthesia (GA). The focus prior to surgery was to avoid carcinoid trigger such as emotional stress and anxiety. In this regard the patient received premedication with Alprazolam 0.5 mg. Standard anesthesia monitoring such as electrocardiogram, noninvasive blood pressure, pulse oximetry and end-tidal CO2 was used. Moreover, anesthesia depth monitoring (NeuroWave®) and cerebral oximetry (INVOS®) were carried out. Before induction a large intravenous catheter was placed, and the radial artery was cannulated. A balanced anesthetic technique was performed with 2 mg of midazolam, 10 mcg of sufentanil, 12 mg of etomidate and 80 mg of 2% lidocaine. To facilitate intubation, 50 mg of rocuronium for neuromuscular blocking was use. To avoid a carcinoid crisis in response to intubation a 50 mcg loading bolus of somatostatin was administered at induction followed by a continuous infusion rate at 100 mcg/h. The intubation was performed with a video laryngoscope (AirTrack®) to avoid excessive stimulation. The maintenance of the anesthesia was done with sevoflurane (End-Tidal Sevoflurane (EtSevo) 1.2–1.5). After induction, a central venous line was placed in the internal jugular vein for volume resuscitation or inotropic support. Our protocol (Fig. 1) agreed that in the event of hypotension, flushing or bronchospasm, an additional bolus of 50 mcg of somatostatin should be administered; after eliminating all other causes, bolus can be repeated 1x/5 min. With the possibility of increased the continuous infusion rate to maximum 300 mcg/h. Treatment of moderate hypotension would be done with phenylephrine or noradrenaline. A dobutamine support was ready to use in the event of refractory collapse. We also took care to exclude all histamine liberating drugs as morphine or atracurium. With regards to the perioperative and postoperative pain management, an ultrasound-guided transversus abdominal plan block with 0.25% levobupivacaine at the dose of 1 mg/kg was placed prior to surgical incision. The total duration of the surgery was 180 min and was well tolerated by the patient. She required two boluses of additional 50 mcg somatostatin each to treat hypotension due to tumor manipulation and 3 boluses of 50 mcg phenylephrine for moderate hypotension. Hemodynamics, heart rate and electrocardiography were continuously registered and remained stable. No intraoperative flushing, oedema or bronchospasm was noticed. At the end of the surgery the patient was awakened, extubated, and transferred to intensive care unit for surveillance. The somatostatin infusion was continued up to 12 h after the surgery. A piritramide pump provided additional postoperative analgesia. The patient did not experience any postoperative complications. She left the intensive care unit 2 days later.

## Discussion

Neuroendocrine tumors are increasingly common in our clinics and their management requires multidisciplinary team approach. Anesthetic management of patients with CaS presents life threatening challenges. The severity of symptoms is not a reliable indicator of the type or severity of the intraoperative response to tumor manipulation [10, 14]. Carcinoid heart disease remains a rare complication and has a significant impact on patient morbidity [2, 8, 9]. The management of carcinoid cardiomyopathy is mainly symptomatic [2, 5, 7]. However, octreotide helps to slow down the development of fibrosis and ultimately heart failure. Once the valvopathy is established, the only curative treatment is valve replacement, a surgery with a high morbidity rate [5, 7, 9]. Depending on the stage of the carcinoid tumour, all potentially resectable tumours (including tumours with limited metastatic disease of the liver) should be completely resected [15]. In view of the irreversible damage to the valvular apparatus and symptomatology, the priority of cardiologic intervention over oncologic intervention must be discussed in multidisciplinary consultation. Some authors [1, 3, 16] report an incidence of carcinoid seizures during anesthesia in patients with carcinoid heart disease ranges from 1 to 8% leading to a significant increase in morbidity and mortality. It is therefore important to take specific precautions to minimize this risk.

Anesthetic key points: Monitoring and appropriate surveillance of the hemodynamics are recommended, including invasive measurement of arterial pressure, cardiac output monitoring by transesophageal echocardiography or pulmonary catheter. The use of octreotide intraoperatively to reduce the incidence of carcinoid crisis is also recommended despite controversial study [17–19]. There is currently no consensus on the administration modality, however the latest ENETS recommendations [16, 20] suggest a bolus of 100mcg at induction followed by a continuous infusion of between 50 and 300 mcg with additional iterative boluses. The maintenance of adequate multimodal anesthesia and analgesia is essential, the use of remifentanil [21], epidural or spinal block [22–24] have been described. Also, monitor the quality and depth of anesthesia by means of BIS or EEG in order to discriminate the different possible etiologies of hypertensive attacks that may mimic a carcinoid crisis [25]. The use of histamine releasing drugs (morphine, pethidine, atracrium) is contraindicated. On the other hand, the use of antihistamines has been described in various studies as prophylaxis [8, 16]. The use of catecholamine as an inotropic or vasopressor support is no longer contraindicated [3, 18, 19] despite previous recommendations [5] – Physiological responses induced by intubation, airway manipulation or hypoxia may be responsible for the onset of carcinoid crisis in up to 60% of case [4], therefore, the use of video laryngoscopy is recommended [3]. Finally, close postoperative monitoring should be recommended [3, 6] in the intermediate or intensive care setting to monitor and treat signs of heart failure or carcinoid syndrome promptly. As the effects of tumor mediators may continue after tumor removal, it is recommended to continue continuous infusion of octreotide during the first 12 h postoperatively.

## Conclusions

Anesthetic management of patients with carcinoid tumor and carcinoid cardiomyopathy can be safely performed with effective hemodynamic monitoring and a good understanding of the pathophysiology. Knowledge and application of a clear institutional algorithm for octreotide administration and multidisciplinary consultation at a referral center are essential for the management of these patients. In our case, the anesthetic key point was to have a management algorithm to anticipate and elucidate the cause of sudden hypotension thus avoiding cardiovascular collapse, using continuous intraoperative somatostatin analog infusion and catecholamine if needed. Regardless, as left CaHD is a complex and rare disease it should be managed in specialized centers with expertise in the field and with the involvement of a multidisciplinary team.

### Supplementary Information

Supplementary Material 1.

## Data Availability

Not applicable.

## Abbreviations

CaHD: Carcinoid heart disease
CaS: Carcinoid syndrome
EtSevo: End-Tidal Sevoflurane
GA: General anesthesia
NYHA: New York Heart association
NETs: Neuroendocrine tumors
PAP: Pulmonary arterial pressure
PET CT: Positron emission tomography
PH: Pulmonary hypertension
TEE: Transesophageal echocardiography
TTE: Transthoracic echocardiography
