# Supplementary material for: Anesthetic key points in a patient with a terminal ileum neuroendocrine tumor and a rare carcinoid left heart disease presented for non-cardiac surgery: case report
Source: BMC Anesthesiol. 2024 Jul 31;24:265. doi: 10.1186/s12871-024-02648-w (PMC11290185; doi:10.1186/s12871-024-02648-w)
Supplement: Supplementary file 1 — Supplementary Material 1. [file 12871_2024_2648_MOESM1_ESM.docx]

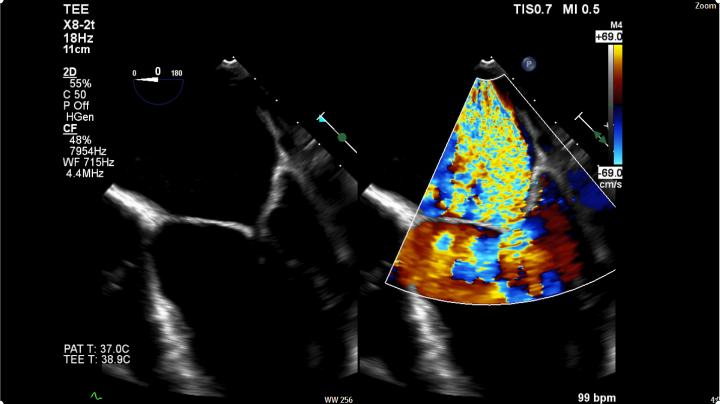

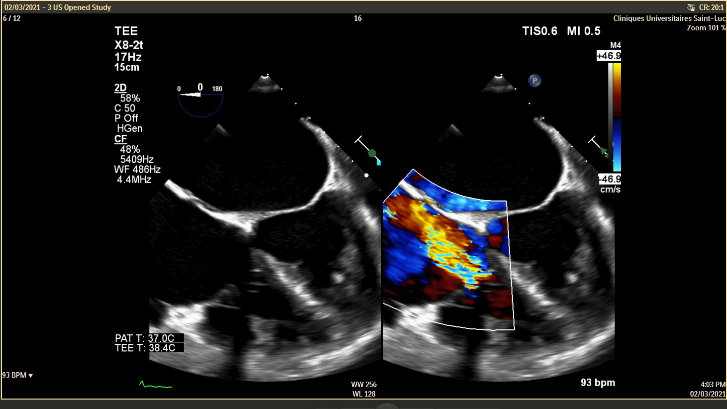
**Annexes – Figure and Images**

C.

B..


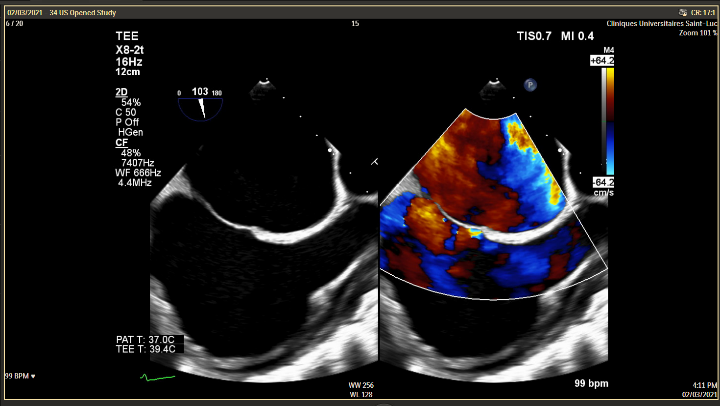

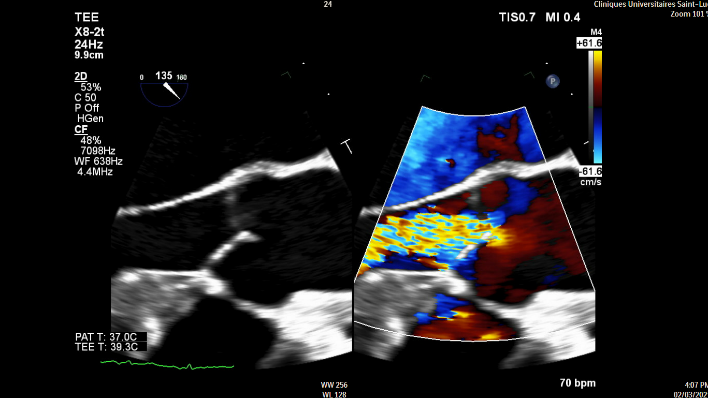


A.

D.

Image 1. Trans-esophageal echocardiographic images. Color Doppler on the different valves. A. Mitral insufficiency with severe mitral regurgitation reaching the atrial roof. B. tricuspid regurgitation. C. severe aortic regurgitation. D. A mid-esophageal bicaval view showing the PFO.
